# How the SARS-CoV-2 Pandemic Period Influenced the Health Status and Determined Changes in Professional Practice among Obstetrics and Gynecology Doctors in Romania

**DOI:** 10.3390/medicina57040325

**Published:** 2021-04-01

**Authors:** Magdalena Iorga, Camelia Soponaru, Răzvan-Vladimir Socolov, Alexandru Cărăuleanu, Demetra-Gabriela Socolov

**Affiliations:** 1 Department of Behavioral Sciences, Faculty of Medicine, “Grigore T. Popa” University of Medicine and Pharmacy Iasi, 700115 Iasi, Romania; magdalena.iorga@umfiasi.ro; 2 Department of Psychology, Faculty of Psychology and Education Sciences, “Alexandru Ioan Cuza” University, 700111 Iasi, Romania; 3 Obstetrics and Gynecology Department, Faculty of Medicine, “Grigore T. Popa” University of Medicine and Pharmacy Iasi, “Elena-Doamna” Obstetrics and Gynecology University Hospital, 700115 Iasi, Romania; razvan.socolov@umfiasi.ro; 4 Obstetrics and Gynecology Department, Faculty of Medicine, “Grigore T. Popa” University of Medicine and Pharmacy Iasi, “Cuza-Voda” Obstetrics and Gynecology University Hospital, 700115 Iasi, Romania; demetra.socolov@umfiasi.ro

**Keywords:** obstetrics and gynecology, physicians, stress, medical practice, psychosomatic symptoms, continuing education, SARS-CoV-2, pandemics, fear of COVID 19

## Abstract

*Background and Objectives*: The beginning of the SARS-Cov-2 pandemic period has had a strong impact on patients’ life, but also on doctors. The main goal of this research is to identify the difficulties related to the professional activity and personal life of obstetrics and gynecology doctors. *Material and Methods*: In total, 94 physicians from a single university center answered to an online questionnaire. Socio-demographic, health, family, and job-related data were collected. Data were processed using SPSS (v.25). *Results*: 7.4% of the doctors were confirmed infected with SARS-Cov-2 during the first 6 months of the pandemic, and 48.94% treated infected patients. Due to the large number of patients, 10.64% of the doctors have had no days-off during the last 6 months, and 22.34% of them have had new medical problems that led them to see a specialist. Seventeen to nineteen percent mentioned an increasing number of working hours and shifts per month due to the pandemic period, more than 10% used pills to cope with work-stress, and 25% of them had sleep disorders along with appetite loss. Extra-protection rules and negative consequences of wearing special equipment were identified: thermal discomfort that caused decreasing resistance and concentration during the surgery (52%), reduced mobility and accuracy of surgical or medical gestures (40%), and intraoperative visibility (47%). Doctors who were working with confirmed pregnant women preferred caesarean section. *Conclusions*: Working under the stress of an infection with SARS-Cov-2 is causing a lot of pressure and determines changes in personal, familial, social, and professional life. Understanding the challenges that ob-gyn doctors are facing will help institutions to better provide support.

## 1. Introduction

Coronaviruses are single-stranded RNA viruses that cause illness ranging in severity from common cold-like symptoms to severe complications that may lead to the death of an individual. The novel coronavirus, SARS-CoV-2, shares common features to two other coronaviruses: severe acute respiratory syndrome coronavirus (SARS-CoV) and the Middle East respiratory syndrome coronavirus (MERS-CoV), but it has caused more cases of illness than were reported for the other two combined [1].

The effects of the SARS-CoV-2 pandemic are complex, with multiple reverberations on social, economic, and psychological levels. In this context, it is important to think about the effects on both mental health and quality of life for the vulnerable categories. Healthcare providers are in the first line of defense for this disease, and every day comes with biological and psychological risks, so a careful evaluation of this parameters is necessary to characterize the overall impact of the new pandemic, and to create effective strategies of management, coping, or protection.

During a pandemic, those with pre-existing mental disorders could experience exacerbations or, regardless of their psychiatric background, individuals may become anxious, helpless, or have a mental breakdown [2]. The most significant psychiatric manifestations range from anxiety, depression, panic attacks, somatic symptoms, and post-traumatic stress disorder (PTSD) to delirium, psychosis, and even suicidality, especially for younger persons, or for those with increased feelings of self-blame [3,4].

Any new disorder induces fear of the unknown, and the lack of information, fake news, or conflicting data can increase the perceived amount of stress by healthcare providers. It is necessary to adapt and to embrace polices that focus on correct dissemination of information, reciprocal support, and community-hospital relationships [5,6,7].

As for the healthcare providers that operate in the field of obstetrics and gynecology, they face numerous daily challenges that may be represented by maternal or fetal complications, material shortcomings, or obstetrical emergencies, which all constitute important stress factors. Even if, theoretically, the risk of contamination from COVID-19 positive patients is lower than for other specialties, they face greater stress, related to the medico-legal responsibility of the medical act in this specialty. For this reason, we consider that the acute stress induced by the COVID-19 pandemic among ob-gyn doctors could have particularities compared to other specialties.

The World Health Organization (WHO) declared 2020 the “Year of the Nurse and the Midwife”, and this action outlined the importance of these health professionals, especially during the COVID-19 pandemic [8].

Bahat et al. [9] evaluated the effects of the COVID-19 pandemic on the physical and mental wellbeing of obstetricians and gynecologists in Turkey through a prospective survey-based study and found that the majority of the respondents (76.7%) reported that they were afraid of coming into contact with pregnant women with confirmed COVID-19, and more than half of them (56.1%) thought that vertical transmission from mother to new-born could be possible. Moreover, in this study, most of obstetricians (82.3%) did not initiate labor earlier during this period, and 51.8% reported that they did not opt for more caesarean deliveries, despite their fear of exposure. 

Even prior to the COVID-19 pandemic, depression, distress, and burnout were higher among ob-gyn physician [10,11,12]. Fear of infection and safety measures determine a shorter and more distant contact with patients and their family members, which has a great negative impact on doctor-patient relationships, communication bond, and the trustful link between doctor and patient [13]. Apart from professionally new experiences, physicians must face personal and family challenges: extra working hours with less physical contact with family members, fear of being infected, fear of passing on the infection to family members, isolation, and less physical activity [14].

In a cross-sectional multicenter study by Vafaei et al. [15], the depression score among obstetrics health care providers was negatively associated with quality of life, while social support had a reinforcing effect during the new pandemic.

Since the first reported case of SARS-CoV-2 infection in Wuhan, China, on 31 December 2019 [16], the world has encountered more than 32 million cases, and almost one million deaths [17]. While the first case of COVID-19 was declared in Wuhan on 31 December 2019, in Romania, the first cases of COVID-19 were reported in the second half of March, followed by the first public health measures and social distance limiting rules.

The Romanian health care system had already faced major material and personal shortcomings before the rise of the new pandemic, so the government, community leaders, and healthcare facilities put in their best efforts to manage this situation. The statistics for the SARS-CoV-2 pandemic in Romania indicate a total of almost 122,000 confirmed cases and 4700 deaths [18,19], which reflects the severity of the situation.

Given the developing circumstances with the new coronavirus, evidence synthesis about mental health outcomes is needed to produce guidance for health care facilities and professionals. The level of risk for healthcare workers from obstetrics and gynecology departments remains unclear. The present study evaluated the opinion of ob-gyn doctors working in Romanian hospitals. And it had several aims: to assess general mental and physical health, to identify changes in clinical practice, to evaluate opinions about the pandemic and the fear about COVID-19 and to identify changes regarding personal, familial, and social behavior. This prospective survey-based study is the first one that evaluates the effects of the SARS-CoV-2 pandemic on Romanian obstetricians.

## 2. Materials and Methods

### 2.1. Participants and Data Collection

The study was conducted between 1 and 30 September 2020. An online test with non-validated items was distributed to obstetrics and gynecology doctors working in two maternities in Iasi. The city of Iasi is the second largest in Romania and is an important university center. One of the two maternity hospitals was designated to accept female patients with a confirmed diagnostic of infection with SARSCoV-2.

At the time of the research, 133 ob-gyn doctors (80 residents, 8 specialists and 45 consultants) were registered in the two university hospitals. The online questionnaire was distributed to all of them. Doctors were informed that they could withdraw from the study whenever they wanted, without consequences. No incentive was given to participants. The inclusion criteria were doctors who had been active in the last six months in one of the maternity hospitals (regardless of gender, age, length of employment, or medical experience) and who returned questionnaires that were fully filled-in and returned before the deadline. 

**Figure 1:**
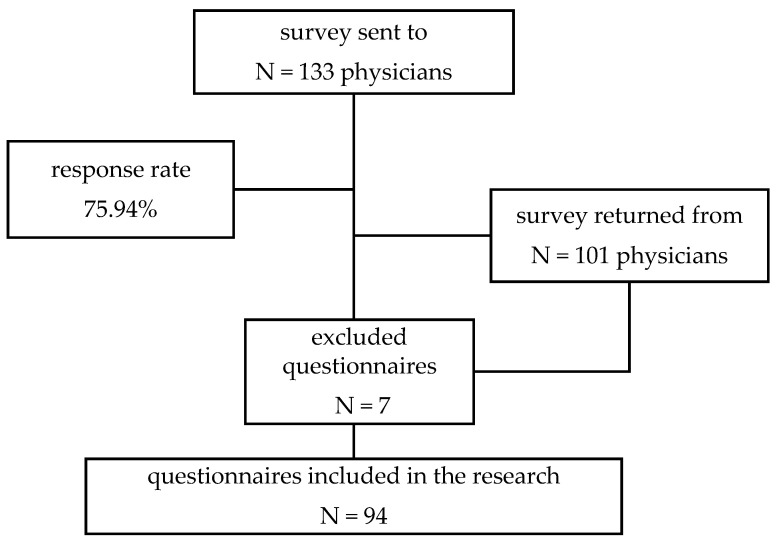
Study profile.

From the total of 133 ob-gyn physicians, 101 respondents filled in the questionnaire; 7 questionnaires were excluded from the research for being incomplete or submitted after 30 September. Finally, 94 questionnaires were included for the analysis of data. Figure 1 provides details of the response rate.

### 2.2. Questionnaire

The questionnaire was constructed especially for this research and was divided into three parts:

- The first part gathered sociodemographic, medical, and family-related data (age, gender, professional level, length of experience, marital status, chronic disease, medication, sleep and appetite self-declared trouble, depressive symptoms, panic attack, self-administration of pills in order to cope with stress, relation with family members and friends during pandemic period, activities during spare time, etc.;
- The second part had several items investigating the aspects related to their professional life during the pandemic period: number of shifts, supplementary measures in order to protect themselves from infection, relationship with patients and their families, practicing caesarean section/natural delivery, dealing with suspected or confirmed patients infected with SARSCoV-2, professional activity changes imposed by the pandemic restrictions, relationship with colleagues, using special equipment when being in contact with a confirmed patient;
- The third part contained items that investigated the opinion of ob-gyn doctors regarding self-perception about the possibility of being infected with SARSCoV-2, fear of infection, and consequences, etc.

### 2.3. Statistical Analysis

All analyses were performed using IBM SPSS Statistics for Windows, version 25. Results for descriptive statistics were expressed as means and standard deviations (SD). Correlation analysis was done using *Spearman* correlation, and for comparative analysis we used the *Independent Sample T-Test*. A *p*-value <0.05 was considered statistically significant.

### 2.4. Ethical Approval

The study was conducted in accordance with the Declaration of Helsinki, and the protocol was approved by “Elena Doamna” University Hospital of Iasi, Romania, with the registration number No. 6843/26.08.2020. Before starting the survey, the participants were informed about the purpose of the research and confidentiality of data. Those who agreed could fill in the on-line questionnaire, and no separate informed consents were obtained or signed.

## 3. Results

### 3.1. Sociodemographic Data

A total of 94 obstetrics and gynecology physicians from two university maternity hospitals participated to the survey. The majority of them were female, and the average age was 36.79 ± 10.81 (with a minimum of 25 and a maximum of 61 years old). Fifty-two of the doctors (55.32%) were married or in a relationship, and 54 (51.06%) were parents. The length of experience was 11.46 ± 10.84 years (with a minimum of 8 months and a maximum of 35 years of experience in the medical field). The distribution of respondents, considering socio-demographic variables, is presented in Table 1.

### 3.2. Health Status and Chronic Disease

Doctors were vey exposed to COVID-19 during pandemic period. Among the questioned ob-gyn physicians, it was revealed that 37 doctors (39.4%) were suspected of being infected and 7 (7.4%) were confirmed. 

Most doctors performed the COVID-19 test at the institution (N = 77, 81.93%), and a few others (N = 9, 9.57%) did their tests in private labs (on personal request). During the first 6 months of the pandemic period, only 8 doctors (8.51%) were not verified for SARS CoV2 infection.

Regarding their health status, 14 (14.89%) presented at least one chronic disease as medical diagnostic. Among the conditions, they mentioned asthma (N = 3), hypertension (N = 5), diabetes (N = 2), autoimmune thyroiditis (N = 2), autoimmune hepatitis (N = 1), chronic rhinitis (N = 1), glaucoma (N = 1), multiple sclerosis (N = 1), chronic migraine (N = 1), vestibular syndrome (N = 1), and liver transplant (N = 1), and 13 (13.83%) were under medical treatment for a chronic disease. 

Doctors were asked about consumption of pills for their chronic disease or self-administrated medicines: energy drinks (including caffeine) (N = 35, 37.23%), antalgic/inflammatories (N = 18, 19.15%), antihypertensives (N = 4, 4.26%), immunosuppressives (N = 3, 3.19%), hypnotic/sedatives at bedtime (N = 3, 3.19%), and 32.98% had not used medicines in the last six months.

We identified that more than one third of respondents used energy drinks (including caffeine. The majority (77.1%) of consumers were women; residents were more prone to this kind of drinks (62.9% of residents, 20% of specialists, and 17.1% of consultants were using caffeine-based drinks)

Physicians were questioned if they had been taking medication to cope with work-related stress in the last 6 months. The answer was positive in 10.64% of respondents.

Doctors were asked about psychosomatic symptoms identified in the last six months. The frequency of answers related to sleep and appetite disorders, medical problems, or even thoughts about changing the job in the health services. Results are presented in Table 2.

We identified that more than half of the respondents self-declared that they had sleep disorders. Among them, 80% were women and 54% were residents. Almost one third of them (30%) use to drink energy drinks, including caffeine). 

### 3.3. Work-Related and Clinical Practice Data

More than a quarter of doctors (26.60%) worked in a support-COVID-19 hospital. Most of them (91.49%) were in contact with patients suspected of being infected, and almost half of doctors (N = 46, 48.94%) had treated infected patients.

Almost two thirds of the doctors declared that their clinical activity had not changed due to the pandemic period, but almost 20% maintained that their activity in the hospital had changed in the last 6 months; 17% mentioned the increasing number of working hours and 19% mentioned the number of shifts per month.

During the pandemic period, in Romania, medical professionals were not allowed to have free days. One item investigated the number of days off in the case of ob-gyn doctors from both hospitals. We identified that 22.30% of doctors had no free days between 31st March and 1st September. The average was 7.29 ± 6.00 free days during the last 6 months. Strong positive correlations were identified between the number of days off and age (r = 0.270, *p* = 0.008), number of children (r = 0.347, *p* = 0.001), and length of experience in the medical field (r = 0.331, *p* = 0.002).

Doctors were asked if they had thought about changing their job in the last six months. For 22 (23.40%), the answer was affirmative (17 women (77.3%) and 5 men (22.70%)). The distribution of answers considering this item is presented in Table 3.

Several items investigated the changes in clinical practice that were imposed by the pandemic situation. Most doctors (89.36%) believed that the pandemic had changed the view of patient interactions and led them to apply new rules and to be more cautious. Items were rated on a 5-point Likert-like scale from 1 to 5 (*never, rarely, sometimes, often, and always*). The frequency of answers is presented in Table 4.

We identified significant statistical differences between doctors who treated (M = 3.09) or did not treat (M = 2.49) confirmed patients. We identified that doctors who were working with confirmed pregnant women preferred caesarean section (*t* = −2.266, *p* = 0.026).

The comparative analysis proved that there were significant differences between genders, category of doctors, and type of institution (COVID-19/non COVID 19 hospitals) regarding pregnant women and childbirth assistance. We identified significant differences on the items that determined if there was any change in the way of tracking pregnant women and childbirth assistance by increasing the percentage of private follow-ups due to the decrease in outpatient activity in the hospital between:

- male (M = 2.45) and female (M = 2.95) doctors (*t* = −2.042, *p* = 0.046), meaning that women, more than men, thought that, during the pandemic, they changed the way of tracking pregnant women and childbirth assistance by increasing the percentage of private follow-up due to the decrease in outpatient activity in the hospital;
- residents (M = 2.84) and specialists (M = 3.38), meaning that specialists, more than residents, directed pregnant women to private clinics for medical services (*t* = −2.145, *p* = 0.042). Additionally, specialists dirrected more patients to private services, compared to consultants (*t* = 2.836, *p* = 0.009, Mspecialists = 3.38, Mconsultants = 2.66);
- doctors who worked in hospital providing care for confirmed patients (M = 3.20) directed patients to private medical clinics more than doctors who were practicing in a non-Covid-19 support hospital, M = 2.72 (*t* = −2.246, *p* = 0.030).

Regarding the preference for caesarean section in order to assure the protection of healthworkers, significant differences were identified (*t* = −2.904, *p* = 0.005) between men (M = 3.17) and women (M = 3.86), and there were also significant differences (*t* = −3.793, *p* = 0.000) between non-Covid hospital physicians (M = 3.41) and those who worked in a Covid-19 support hospital (M = 4.32). So, our results showed that there is a significant difference between genders when it comes to caesarean intervention with the purpose of maximizing protection against coronavirus. We identified that women (M = 3.86), more than men (M = 3.7), preferred to provide C-sections (*t* = −2.904, *p* = 0.005). We also found that there is a significant difference (*t* = −3.793, *p* = 0.000) between doctors working in a hospital that provide medical services for patients infected with COVID-19 (M = 4.32) and those who preferred more to have C-sections in confirmed pregnant women compared to those who worked in a hospital that does not accept confirmed pregnant women (M = 3.41).

We identified a negative correlation with age (*t* = −0.216, *p* = 0.036); the older the doctor, the more caesarean section was preferred in pregnant women suspected or confirmed with COVID-19, due to the patients’ stress related to birth.

Given the fact that communication with the relatives of patients has decreased compared to the previous period before the pandemic, the doctors were asked to what extent they considered that the stress of the medical staff had changed because of this. More than half (N = 48, 51.06%) maintained that there was an increased level of job-related stress, one third of them (N = 30, 31.91%) considered that there was no change regarding the level of job stress, and the rest (N = 16, 17.02%) felt that stress at work had diminished in the last 6 months.

### 3.4. Protection Measures and New Behaviours against Infection

Doctors were asked about the effect of the new rules applied to diminish the risk of infection, and they were asked to self-rate their level of fear regarding certain aspects. The answers were distributed on a 5-points Likert scale, from 1 (*never*) to 5 (*always*), and the results are presented in Table 5.

More than half of the doctors (65.96%) declared that they had short periods in which they had not lived at home or had not visited their families in order to protect them from possible infection. 

Additionally, 87.23% of respondents maintained that they applied additional protection measures when getting home in order to secure their home (they undressed at the door, took a shower immediately, disinfected themselves etc.).

### 3.5. Daily Life, Social and Leisure Activities

We found that, in the last 6 months, more than a third of the ob-gyn physicians (N = 34, 36.17%) spent more time with their family, and a quarter of them (N = 25, 26.60%) had increased their physical activity during the previous 6 months. Among the most frequent activities that doctors spent their free time doing during previous 6 months were: watching TV (28.72%), reading (11.7%), jogging, and cooking (12%). 

Three quarters of doctors (73.40%) declared that the pandemic had changed the view of the profession rules; for 71.28%, the pandemic had changed the hierarchy of their priorities in life, and 65.96% maintained that this last period had changed their vision about life and the world. 

Socializing had been another problem from the beginning of the pandemic. Respondents were questioned about changes in their relationships with family members, colleagues, and friends. The most affected relationships were those with friends, and the less affected were relationships with colleagues at work. The distribution of answers for these items is presented in Table 6. 

### 3.6. Impact of the Pandemic Period on ongoing Education, Organization Rules

Ongoing medical education was also affected by the restrictions imposed by the pandemic; all courses were provided online. Almost three quarters of the doctors (74.47%) maintained that they had online lectures, and 68.09% stated that it was exceedingly difficult to adjust to ongoing online medical development.

Subjects also appreciated what the advantages of online education were, compared to onsite education. Among the advantages, doctors mentioned that online course reduced related costs (18.09% of respondents), it streamlined the integration of online medical education into their daily schedule (40.43%), the course content was always available (34.04%), it helped to improve practical ability by respecting social distancing (2.1%), and it was considered much better than onsite courses because it allowed socializing with colleagues from other hospitals and counties (5.32%).

A series of items were formulated to identify the changes in clinical activity, protocol, and organizational rules. Some others pointed out the changes regarding relationships with colleagues and how clinical activity was affected by social distance rules. Answers were credited using a 5-points Likert scale, from 1 (*never*) to 5 (*always*). The frequency of answers is presented in Table 7.

Working in a hospital that provided medical care to women who had been confirmed with COVID-19 needed more rules to protect medical professionals. The results showed a significant difference between COVID-19 and non-COVID-19 maternity hospitals, in the sense that doctors working in non-COVID-19 hospitals noticed, to a greater extent, the fact that, starting from the beginning of pandemic period:

- on-call reports were cancelled (M = 4.87, *t* = 5.137, *p* = 0.000) compared to subjects working in COVID-19 hospitals (M = 3.56);
- on-call reports were kept online (M = 3, *t* = 4.974, *p* = 0.000) compared to subjects working in COVID-19 hospitals (M = 1.20);
- more professional online communication groups/networks were created at the hospital, clinic, residents, and ward level (M = 4.54, *t* = 3.052, *p* = 0.004) compared to subjects working in COVID-19 hospitals (M = 3.88);
- there were more professional discussions on the phone or online than face-to-face meetings (M = 3.91, *t* = 2.295, *p* = 0.028) compared to subjects working in COVID-19 hospitals (M = 3.36);
- more medical education was conducted online (M = 4.28, *t* = 2.494, *p* = 0.014)) compared to subjects working in COVID-19 hospitals (M = 3.60).

### 3.7. Ethical Concerns and Malpractice

Obstetricians were asked if they thought their specialty could be adapted to online consultations due to pandemic restrictions. More than one third of them (32.96%) did not agree to have online medical consultations with pregnant patients. Out of all questioned doctors, only a quarter (N = 23, 24.47%) maintained that they offered online advice to their patients. 

Among the reasons why they did not give online consultations, ob-gyn doctors mentioned risk of malpractice–misdiagnosis (N = 48, 51.06%), lack of legal or professional regulation (N = 41, 43.61%), lack of access to the online system (N = 10, 9.4%), and lack of remuneration (N = 6, 6.38%).

### 3.8. Fear of Infection, Hospitalization, and Disease Side-Effects

Participants were asked about their fear of being infected by their patients and passing on the disease to their family members. Additionally, due to the paucity of scientific information regarding the recovery, side-effects, and impact on healthy people, some items focused on identifying their fear regarding the medication and possible sequelae. The answers were credited using a 5-points Likert scale, from 1 (*never*) to 5 (*always*). The results are presented in Table 8.

Comparative analysis revealed that there were significant differences regarding gender, the presence of a chronic disease, marital status, and doctors who chose to stay away from family members when it came to fear of treatment for infection and its consequences. We identified that:

- women were more afraid of anti-COVID-19 medication and its secondary effects than men (M_women_ = 3.55, M_men_ = 2.83, *t* = −2.555, *p* = 0.013);
- doctors who were married were more afraid of interrupting their clinical activity in case of infection (*t* = −2.201, *p* = 0.03, M = 3.92) compared to single people (M = 3.43);
- doctors with no children (M = 4.17) presented a significantly higher level of fear of transmitting the infection (*t* = 1.757, *p* = 0.003) compared to doctors who were parents (M = 3.52).

Several comparative differences were identified between doctors who were diagnosed with a chronic disease compared to those who did not have a long-term disease in terms of:

- *fear of anti-Covid medication and its side effects* (*t* = −2.070, *p* = 0.05) in the sense that those with chronic diseases had a greater fear (M = 3.93) compared to those without chronic diseases (M = 3.23);
- *fear/risk of death* (*t* = −2.904, *p* = 0.005) in the sense that those with chronic diseases had a greater fear (M = 3.93) compared to those without chronic diseases (M = 2.89);
- *fear of lung disease* (*t* = −2.549, *p* = 0.012) in the sense that those with chronic diseases had a greater fear (M = 4.29) compared to those without chronic diseases (M = 3.51);
- *fear of extrapulmonary pathology* (*t* = −2.707, *p* = 0.013) in the sense that those with chronic diseases had a greater fear (M = 4) compared to those without chronic diseases (M = 3.28);
- *fear of distant sequelae* (*t* = −2.890, *p* = 0.01) in the sense that those with chronic diseases had a greater fear (M = 4.21) compared to those without chronic diseases (M = 3.45).

Additionally, considering the decision to leave home for short periods of time in order to protect their families, we identified that there were significant differences between:

- doctors who decided to protect their families by living elsewhere or restricting visits and therefore presented a greater fear of transmitting the disease to colleagues, and the fact that he/she would be accused of this and stigmatized/marginalized collectively. (M = 3.13, t = −2.366, p = 0.013) compared to those who did not take these measures (M = 2.50),
- those who protected their families by living elsewhere or restricting visits had a greater *fear of transmitting the disease to their family members* (M = 4.05, *t* = −2.498, *p* = 0.016) compared to those who did not take these measures (M = 3.44)
- doctors who protected their families by living elsewhere or restricting visits had a greater *fear of anti-Covid medication* (M = 3.55, *t* = −2.352, *p* = 0.021) compared to those who did not take these measures (M = 2.91)

## 4. Discussion

Few studies have focused on the impact of COVID-19 on the psychological, physical, and professional life of obstetrics and gynecology physicians. The present research showed that a lot of changes affected physicians’ lives, on all levels. Their personal life had to adjust to the new rules of isolation, social distance, and self-care. The relationship with the family was disturbed due to concerns about not transmitting a possible infection. We identified that most doctors (87.23%) applied additional protective measures when getting home. More than half of the doctors (65.96%) declared that they had short periods in which they had not lived at home or had not visited their families in order to protect them from possible infection, and a large majority maintained that they applied additional protective measures when getting home. In a similar research on ob-gyn doctors in Turkey, Bahat et al. (2020) found that 74.4% of ob-gyn doctors stated that they were afraid of getting sick, 64.8% reported that they had fallen into despair at times because of the pandemic, 66.5% stated that their family lives had been affected, and 72.4% had started living separately from their families because of the pandemic [9].

Another identified problem was the permanent involvement into clinical practice. We identified that almost a quarter of the doctors had no free days during the first six months of the SARS-CoV-2 pandemic, and more than a half maintained that there was an increased level of job stress. Almost a quarter of them (majority women) declared that they had thoughts of changing their jobs. The use of pills to cope with stress and the intake of energy drinks was found to be frequent among the respondents (37.23% for energy drink intake, including caffeine), with women consuming more frequently than men and residents consuming more frequently compared to specialists or consultants.

Sleep disturbance, anxiety, and fear of contagion were also identified by a few studies conducted among doctors during the first six months of the pandemic period. In the study of Pappa et al. (2020), it was found that at least one in five healthcare professionals reported symptoms of depression and anxiety, and almost four in ten healthcare workers experienced sleeping difficulties and/or insomnia. [20] Additionally, the prevalence rate of anxiety and depression in female healthcare workers and nursing staff obtained a high scores for anxiety. Zhang et al. found that 36.1% of doctors suffered from insomnia symptoms during the beginning of the pandemics [21]. Doctors working in isolation units and worrying about being infected were more prone to experiencing sleep disturbance. We identified that sleeping problems were present in half of the respondents (80% were women and 54% were residents, which is in congruency with the presented results). Appetite loss was also identified among psychosomatic problems in a quarter of the respondents.

The results of our study proved that doctors were afraid of passing on the infection to their family members, of being hospitalized, or having possible long-term sequels. Talevi et al. [5] showed that most individuals experienced considerable psychological distress during the initial stage of the COVID-19 outbreak in terms of anxiety, depression, and post-traumatic symptoms, and that the symptoms’ severity was evaluated as mild to moderate. Moreover, in the early days of the outbreak, a survey found that 53.8% of respondents rated the psychological impact of the outbreak as moderate or severe, 16.5% reported moderate to severe depressive symptoms, 28.8% reported moderate to severe anxiety symptoms, and 8.1% reported moderate to severe stress levels [6].

Medical practice had to adjust to new rules (institutional organization, doctor-patient relationships, ethical and legal rights, etc. We identified that doctors who were working with confirmed pregnant women preferred a caesarean delivery instead of a normal one, with the purpose of avoiding infection.

The insufficient equipment problem that doctors had to deal with in the beginning of the pandemic period was a strong source of stress. Medical staff had to be equipped with full-body protective equipment under the negative pressure of providing medical care for pregnant women during medical care or the delivery process. We identified that more than half of the doctors strongly agreed that the suits warmed them too much, and that the thermal discomfort decreased their resistance and concentration during surgery (52%), reduced the mobility and the accuracy of surgical or medical gestures (40%), and caused intraoperative visibility (47%).

To avoid being infected while removing the protective equipment, ob-gyn doctors cannot eat, drink, or use the bathroom for several hours. Dehydration and excessive sweating, cystitis, and a rash were identified by Marjanovic et al. (2006) on medical staff during the previous SARS pandemic [22]. Doctors working with confirmed patients were more prone to have sleep disturbance and high levels of stress. 

Our study revealed that the doctors had difficulty in counselling pregnant women, especially patients who missed mandatory tests in the prenatal consultation, due to their fear of exposing themselves to possible infection during pregnancy. The results are in congruency with Mappa et al., who showed that the COVID-19 pandemic induced a doubling of the number of women who reached an abnormal level of anxiety [23]. The authors identified that pregnant women were fearful that COVID-19 could induce fetal structural anomalies (in 47% of investigated pregnant women), fetal growth restriction (in the case of 65% of patients), and preterm birth (in 51% of the women). Derya et al. [24] showed that the tele-education offered to the pregnant women on pregnancy and birth planning during COVID-19 decreased their prenatal distress and anxiety levels.

We identified that a quarter of doctors offered online consultations to their pregnant patients. The study of Derya et al. [24] proved that tele-medicine considerably improved patients’ mental health during pregnancy. Using a control group, the authors showed that pregnant women who received counselling scored lower for anxiety, fear of giving birth, and worries of bearing a physically or mentally handicapped child.

We identified that there was a permanent concern regarding pandemic-associated malpractice cases. Being afraid of the risk of malpractice—misdiagnosis (51.06%) and the lack of legal or professional regulation (43.61%)—were among the most challenging problems during the beginning of the pandemic. Similar results were obtained by other studies with higher rates; in a study conducted on ob-gyn doctors in Turkey, it was found that 82.6% of doctors declared that they were worried about malpractice during the pandemic period [9].

Our study showed that three quarters of the doctors (73.40%) declared that the pandemic had changed their view of the rules of the profession. The results proved that clinical activity changed after the COVID-19 pandemic compared to the period before the pandemic; most doctors (89.36%) believed that the pandemic had changed their view of patient interactions and led them to apply new rules and to be more cautious. The results are congruent with those obtained by Bahat el al. [9], who showed that many ob-gyn doctors (81.5%) believed that their workload increased significantly compared to the period before the pandemic. Additionally, the doctors declared that they were concerned about their private office work being interrupted in the case of infection. The results are similar to those identified in a study conducted at the beginning of the SARScoV2 pandemic in the United States. A few weeks into the pandemic, the Medical Group Management Association found that COVID-19 was having a negative financial effect on 97% of the 724 medical practices [25].

We identified that the older the doctor, the more a caesarean section was preferred in pregnant women suspected or confirmed with COVID-19, due to the patients’ stress related to the birth. Additionally, we found that doctors who were working with confirmed pregnant women preferred caesarean section. The results are congruent with those identified by Bahat et al. [9], who identified that less than a half of ob-gyn reported that they did opt for more caesarean deliveries despite their fear of exposure.

### 4.1. Strength and Limitations of the Study

The most important strength of the research is due to the fact that this is the first study on obstetrics and gynecology doctors working under the pressure of SARS-CoV-2 infection in Europe. Secondly, the study provides a large amount of information relating to psychological and somatic health, clinical practice, behaviors to diminish the risk of infection, new daily habits practice in order to diminish the risk of self-infection and infecting the members of the family, and new institutional rules and methods to deal with daily and job-related stress.

The first limitation of the study is represented by the limited territorial research. The respondents were selected from a single university city center, both maternity hospitals being university centers. Because of this limitation, the results cannot be generalized for all obstetrics and gynecology physicians. Secondly, we must take into consideration the fact that, in Romania, ob-gyn doctors provide medical support in both types of delivery (C-section or normal delivery), so greater risks and concerns regarding infection and job stress could be generalized only for Romanian doctors. Thirdly, the study was conducted after the first six months of the COVID-19 pandemic, so the results reflected the practice of preventive measures applied during this period, congruent with the guidelines in the medical field, precisely because, in the very first months of the pandemic, there were insufficient scientific data concerning the transmission of the infection from mother to fetus/new-born.

### 4.2. Reflections and Planning

The present study shows that the COVID 19 pandemic had negative psycho-somatic effects and led to changes in lifestyle, interaction with family members and friends, the patient’s medical approach, and the adoption of new practices among obstetrics and gynecology physicians. These results prove the need for the adoption of new strategies regarding both the doctors’ health (psychological assistance to treat high levels of stress, overwork, burnout, depression, or fear of infection due to the long negative effect and the considerable period of the pandemic), identified, in general, among healthcare workers during this period [26] and patients’ health, by adopting new protocol guidelines in the field of obstetrics and gynecology, focusing especially on female gender and resident doctors. Secondly, the need for new institutional rules to decrease the risk of infection has imposed new methods of communication (online meetings, tele-medicine, online education) that should be regulated to eliminate the risk for malpractice as much as possible [27]. The psychological assistance of healthcare workers (such as psychological counselling, cognitive-behavioral therapy, or Balint groups for doctors) must be a priority for medical institutions and health-policy makers, due do the fact that the pandemic seemed to be longer than expected and the negative impact on mental health could lead to chronic consequences.

## 5. Conclusions

Even if they were not in the front line fight against the COVID 19 pandemic, working under the stress that they could be at risk of infection, or infecting patients or family members, put a lot of pressure on obstetrics and gynecology doctors, which had a great impact on their physical and mental health. Understanding the challenges that ob-gyn doctors were facing, especially at the beginning of the pandemic, will help institutions to better provide psychological and organizational support, considering their gender, marital status, parental role, age, comorbidities, and type of institutions, and to highlight guidelines to help them cope better with pandemic challenges.

## Figures and Tables

**Table 1:** The distribution of respondents considering the gender and professional level^.^

Socio-Demographic Variables	N(%)/M± ^1^
Age	36.79 ± 10.81
Length of employment (years)	11.46 ± 10.84
Gender	
Male	65 (69.15%)
Female	29 (30.85%)
Marital status	
Single	42 (44.68%)
In relationship	52 (55.32%)
Having children	
yes	54 (51.06%)
no	40 (48.94%)
Level of specialization	
Resident	49 (52.13%)
Male	11 (11.70%)
Female	38 (40.43%)
Specialist	13 (13.83%)
Male	3 (3.10%)
Female	10 (10.64%)
Consultant	32 (24.04%)
Male	15 (15.96%)
Female	17 (18.09%)
Working institution	
COVID-19 maternal services	25 (26.60%)
Non- COVID-19 maternal services	69 (73.40%)

^1^ Number of answers (N) and percentage (%), Means and standard deviations (M±).

**Table 2:** The frequency of answers considering the psychosomatic symptoms in the last 6 months.

Items	Yes (N, %) ^1^	Women	Men
*Have you had any medical problems in the last 6 months that would lead you to see a doctor?*	21 (22.34%)	18 (85.7%)	3 (14.3%)
*Have you had any sleep disorders in the last 6 months?*	50 (53.19%)	40 (80%)	10 (20%)
*Have you had any appetite disorders in the last 6 months?*	32 (34.04%)	26 (81.30%)	6 (18.80%)
*Have you had depressive disorders in the last 6 months?*	17 (18.09%)	15 (88.20%)	2 (11.80%)
*Have you had panic attacks in the last 6 months?*	21 (22.34%)	21 (100%)	0 (0%)
*Have you used the psychological services of the unit in the last 6 months?*	0 (0%)	0 (0%)	0 (0%)

^1^ Number of answers (N) and percentage (%).

**Table 3:** Distribution of answer regarding changing job, considering the level of specialization.

Item *Have You Thought about Changing Your Job in the Last 6 Months?*	Yes ^1^	No ^1^
Residents (N = 49)	10 (20.40%)	39 (79.60%)
Specialists (N = 13)	5 (38.47%)	8 (61.53%)
Consultants (N = 32)	7 (21.87%)	25 (78.13%)

^1^ Number of answers (N) and percentage (%).

**Table 4:** The frequency of items investigating the changes in clinical practice.

Items	N (%) ^1^
During the pandemic, we followed up pregnant women who missed mandatory tests in the prenatal consultation (double genetic test, 12–14 weeks; TTGO, 24–28 weeks; morphological ultrasound, 19–24 weeks, etc.) due to fear of infection with COVID-19.	2.85 ± 0.91
never	7 (7.40%)
rarely	26 (27.70%)
sometimes	35 (37.20%)
often	26 (27.70%)
always	0
During the pandemic, we changed the way of tracking pregnant women and childbirth assistance by increasing the percentage of private follow-up due to the decrease in outpatient activity in the hospital.	2.79 ± 1.07
never	15 (16.0%)
rarely	21 (22.30%)
sometimes	27 (28.70%)
often	30 (31.90%)
always	1 (1.10%)
During the pandemic, caesarean section was preferred for pregnant women suspected or confirmed with COVID-19 to ensure the protection of staff, by reducing the duration of exposure (4–12h, natural birth vs. 1–2h, caesarean section).	3.65 ± 1.10
never	6 (6.40%)
rarely	9 (9.60%)
sometimes	16 (17.0%)
often	44 (46.80%)
always	19 (20.20%)
During the pandemic, caesarean section was preferred in pregnant women suspected or confirmed with COVID-19 due to the specific maternal risk associated with it (dyspnoea due to maternal respiratory phenomena that would make expulsion difficult).	3.65 ± 1.10
never	15 (16.0%)
rarely	16 (17.0%)
sometimes	18 19.10%)
often	33 (35.10%)
always	12 (12.80%)
During the pandemic, caesarean section was preferred in pregnant women suspected or confirmed with COVID-19 because there are serious cases in which the pregnancy had to be completed immediately.	2.79 ± 1.30
never	21 (22.30%)
rarely	18 (19.10%)
sometimes	25 (26.60%)
often	20 (21.30%)
always	10 (10.60%)
During the pandemic, caesarean section was preferred in pregnant women suspected or confirmed with COVID-19 due to the patient’s stress related to childbirth.	2.27 ± 1.15
never	33 (35.10%)
rarely	22 (23.40%)
sometimes	21 (22.30%)
often	17 (18.10%)
always	1 (1.10%)
In the conditions in which communication with the relatives was reduced (compared to the non-COVID-19 period), additional stress was identified in the patient (with panic phenomena, lack of cooperation, aggression).	2.99 ± 0.88
never	5 (5.30%)
rarely	20 (21.3%0)
sometimes	42 (44.7%0)
often	25 (26.6%0)
always	2 (2.10%)
Given the reduced communication with the relatives (compared to the non-COVID-19 period), additional stress of the relatives was identified (with panic phenomena, lack of cooperation, aggression).	2.16 ± 0.67
never	4 (4.30%)
rarely	20 (21.3%)
sometimes	32 (34.0%)
often	35 (37.20%)
always	3 (3.20%)

^1^ Number of answers (N) and percentage (%).

**Table 5:** The distribution of answers to the items investigating the protection measures against infection.

Items	M ± st.dev/
	N (%) ^1^
I used the anti-Covid suit for consultations/pregnant ultrasounds/suspicious/COVID-19 positive postpartum women	3.90 ± 1.31
never	7 (7.40%)
rarely	9 (9.60%)
sometimes	16 (17.0%)
often	16 (17.0%)
always	46 (48.90%)
I used anti-Covid clothing during surgery/birth assistance/ICU manoeuvers/resuscitation manoeuvers in the new-born	3.54 ± 1.43
never	14 (14.90%)
rarely	9 (9.60%)
sometimes	16 (17.0%)
often	22 (23.40%)
always	33 (35.10%)
I consider that wearing a protective suit reduces my intraoperative visibility	4.21 ± 0.94
Never agree	−2.1
Rarely agree	3 (3.20%)
Sometimes agree	16 (17.0%)
Often agree	29 (30.90%)
Always agree	44 (46.80%)
I consider that wearing a protective suit reduces my mobility and the accuracy of surgical or medical gestures.	3.94 ± 1.15
Never agree	4 (4.30%)
Rarely agree	9 (9.60%)
Sometimes agree	16 (17.0%)
Often agree	28 (29.80%)
Always agree	37 (39.40%)
I consider that wearing a protective suit warms me too much, and the thermal discomfort decreases my resistance and concentration during surgery.	4.19 ± 1.05
Never agree	3 (3.2%)
Rarely agree	3 (3.2%)
Sometimes agree	15 (16.0%)
Often agree	24 (25.5%)
Always agree	49 (52.1%)
I consider that wearing the FP2/3 mask causes hypoxia, reducing the power of concentration during the surgical gesture/intervention.	3.28 ± 1.21
Never agree	10 1(0.6%)
Rarely agree	13 (13.8%)
Sometimes agree	29 (30.9%)
Often agree	27 (28.7%)
Always agree	15 (16.0%)
I consider that wearing the suit produces unpleasant effects for me: fainting, etc.	3.19 ± 1.37
Never agree	13 (13.8%)
Rarely agree	22 (23.4%)
Sometimes agree	19 (20.2%)
Often agree	20 (21.3%)
Always agree	20 (21.3%)
I consider that wearing the suit causes me difficulties related to physiological needs that must be repressed until the end of the intervention.	3.11 ± 1.34
Never agree	13 (13.8%)
Rarely agree	23 (24.5%)
Sometimes agree	23 (24.5%)
Often agree	17 (18.1%)
Always agree	18 (19.1%)
I am afraid that the protective equipment provided does not protect me enough.	2.51 ± 1.08
never	18 (19.1%)
rarely	32 (34.0%)
sometimes	28 (29.8%)
often	10 (10.6%)
always	6 (6.4%)
I am afraid of contamination when undressing, even if we have the appropriate protective equipment.	3.14 ± 1.23
never	8 (8.5%)
rarely	25 (26.6%)
sometimes	27 (28.7%)
often	19 (20.2%)
always	15 (16.0%)
I am scared because we do not have complete equipment.	2.37±1.164
never	25 (26.6%)
rarely	30 (31.9%)
sometimes	24 (25.5%)
often	7 (7.4%)
always	8 (8.5%)
I am afraid because we have complete equipment, but it is not adequate, although it is complete.	2.33 ± 1.15
never	28 (29.80%)
rarely	28 (29.80%)
sometimes	22 (23.40%)
often	10 (10.60%)
always	6 (6.40%)

^1^ Number of answers (N) and percentage (%), Means (M) and standard deviations (±).

**Table 6:** Relationship with family members, friends, and colleagues—in the last 6 months ^1^.

Items	Developed in the Same Way N (%)	Have Deteriorated N (%)	Have Improved N (%)	I Do Not Know N (%)
In the last 6 months, family relationships:	63 (67)	10 (10.6)	11 (11.7)	10 (10.6)
In the last 6 months, relationships with friends:	45 (47.9)	31 (33)	8 (8.5)	10 (10.6)
In the last 6 months, relations with colleagues:	70 (74.5)	13 (13.8)	5(5.3)	6 (6.4)

^1^ Number of answers (N) and percentage (%).

**Table 7:** Relationship with superiors and colleagues—in the last 6 months.

Items	M ± St.dev. ^1^
I noticed that, during the pandemic, socializing with colleagues changed.	3.16 ± 1.26
never	13 (13.80%)
rarely	15 (16.0%)
sometimes	24 (25.50%)
often	28 (29.80%)
always	14 (14.90%)
I noticed that, during the pandemic, the morning handovers were cancelled.	4.47 ± 1.17
never	7 (7.40%)
rarely	2 (2.10%)
sometimes	4 (4.30%)
often	7 (7.40%)
always	74 (78.70%)
I noticed that, during the pandemic, the morning handovers were kept online.	2.52 ± 1.78
never	49 (52.1%)
rarely	7 (7.40%)
sometimes	6 (6.40%)
often	4 (4.30%)
always	28 (29.80%)
I noticed that, during the pandemic, extra professional discussions in the locker rooms/hospital lobby/cafe were rarer.	3.29 ± 1.26
never	10 (10.60%)
rarely	14 (14.90%)
sometimes	29 (30.90%)
often	20 (21.30%)
always	21 (22.30%)
I noticed that, during the pandemic, the visits to the sector were made with a smaller number of colleagues or residents.	3.80 ± 1.20
never	5 (5.30%)
rarely	10 (10.60%)
sometimes	18 (19.10%)
often	26 (27.70%)
always	35 (37.20%)
I noticed that, during the pandemic, professional online communication groups/networks were created at hospital, clinic, by residents, and in ward.	4.36 ± 1.02
never	3 (3.20%)
rarely	3 (3.20%)
sometimes	11 (11.70%)
often	17 (18.10%)
always	60 (63.80%)
I noticed that, during the pandemic, there were professional discussions over the phone or online rather than face to face.	3.76 ± 0.97
never	2 (2.10%)
rarely	6 (6.40%)
sometimes	28 (29.80%)
often	34 (36.20%)
always	24 (25.50%)
I believe that the reduction in hospital activity means that residents have fewer cases to deal with personally.	4.28 ± 1.00
never	1 (1.10%)
rarely	6 (6.40%)
sometimes	13 (13.80%)
often	19 (20.20%)
always	55 (58.50%)

^1^ Means, number of answers (N) and percentage (%).

**Table 8:** The distribution of questions regarding the fear of contagion, hospitalization, and infection side-effects^1^.

Items	1	2	3	4	5	M ± st
*I am afraid I am getting infected with COVID-19 from the pregnant patients*	8 (8.50%)	23 (24.50%)	32 (34.0%)	23 (24.50%)	8 (8.50%)	3.00 ± 1.08
*I am afraid that I will transmit the disease to colleagues, and I will be accused of this and stigmatized/marginalized collectively.*	15 (16.0%)	17 (18.10%)	30 (31.90%)	25 (26.60%)	7 (7.40%)	2.91 ± 1.17
*I am afraid I will pass on the disease to family members.*	2 (2.10%)	9 (9.60%)	25 (26.60%)	25 (26.60%)	33 (35.10%)	3.84 ± 1.09
*I am afraid of anti-COVID-19 medication and its side effects.*	9 (9.60%)	21 (22.30%)	18 (19.10%)	22 (23.40%)	24 (25.50%)	3.33 ± 1.33
*I am afraid of the precarious hospital conditions I might have if I had tested positive for COVID-19.*	4 (4.30%)	1 (1.10%)	13 (13.80%)	31 (33.0%)	45 (47.90%)	4.22 ± 0.98
*I am afraid that, in case of infection, I will have to interrupt my professional activity in the state/private system for a longer period.*	4 (4.30%)	8 (8.50%)	28 (29.80%)	26 (27.70%)	28 (29.80%)	3.70 ± 1.11
*I am afraid that, in case of infection, there is a risk that I will be accused by patients of getting COVID-19 infection from me.*	22 (23.40%)	24 (25.50%)	20 (21.30%)	19 (20.20%)	9 (9.60%)	2.67 ± 1.29
*I am afraid that, in case of infection, there is a risk of death.*	10 (10.60%)	27 (28.70%)	24 (25.50%)	15 (16.0%)	18 (19.10%)	3.04 ± 1.28
*I am afraid that, in case of infection, there is a risk of lung pathology.*	2 (2.10%)	12 (12.80%)	30 (31.90%)	25 (26.60%)	25 (26.60%)	3.63 ± 1.07
*I am afraid that, in case of infection, there is a risk of extra pulmonary pathology.*	2 (2.10%)	23 (24.50%)	27 (28.70%)	21 (22.30%)	21 (22.30%)	3.38 ± 1.14
*I am afraid that, in case of infection, there is a risk of remote sequelae.*	0	18 (19.10%)	30 (31.90%)	21 (22.30%)	25 (26.60%)	3.56 ± 1.08

^1^ Number of answers (N) and percentage (%), means and standard deviations (M±).

## Data Availability

The data presented in this study are available on request from the corresponding author.

## References

[1] Rasmussen S.A., Smulian J.C., Lednicky J.A., Wen T.S., Jamieson D.J. (2020). Coronavirus Disease 2019 (COVID-19) and pregnancy: What obstetricians need to know. Am. J. Obstet. Gynecol..

[2] Hall R.C., Hall R.C., Chapman M.J. (2008). The 1995 Kikwit Ebola outbreak: Lessons hospitals and physicians can apply to future viral epidemics. Gen. Hosp. Psychiatry.

[3] Tucci V., Moukaddam N., Meadows J., Shah S., Galwankar S.C., Kapur G.B. (2017). The Forgotten Plague: Psychiatric Manifestations of Ebola, Zika, and Emerging Infectious Diseases. J. Glob. Infect. Dis..

[4] Sim K., Huak Chan Y., Chong P.N., Chua H.C., Wen Soon S. (2010). Psychosocial and coping responses within the community health care setting towards a national outbreak of an infectious disease. J. Psychosom Res..

[5] Talevi D., Socci V., Carai M., Carnaghi G., Faleri S., Trebbi E., di Bernardo A., Capelli F., Pacitti F. (2020). Mental health outcomes of the CoViD-19 pandemic. Riv. Psichiatr..

[6] Wang C., Pan R., Wan X., Tan Y., Xu L., Ho C.S., Ho R.C. (2020). Immediate Psychological Responses and Associated Factors during the Initial Stage of the 2019 Coronavirus Disease (COVID-19) Epidemic among the General Population in China. Int. J. Environ. Res. Public Health.

[7] Kang L., Li Y., Hu S., Chen M., Yang C., Yang B.X., Wang Y., Hu J., Lai J., Ma X. (2020). The mental health of medical workers in Wuhan, China dealing with the 2019 novel coronavirus. Lancet Psychiatry.

[8] World Health Organization WHO Director-General’s Statement on IHR Emergency Committee on Novel Coronavirus (2019-nCoV); WHO: Geneva, Switzerland. https://www.who.int/dg/speeches/de-tail/who-director-general-s-statement-on-ihr-emergency-committee-on-novel-coronavirus-(2019-ncov).

[9] Yalçın Bahat P., Aldıkaçtıoğlu Talmaç M., Bestel A., Topbas Selcuki N.F., Karadeniz O., Polat I. (2020). Evaluating the effects of the COVID-19 pandemic on the physical and mental well-being of obstetricians and gynecologists in Turkey. Int J. Gynaecol. Obstet..

[10] Iorga M., Socolov V., Muraru D., Dirtu C., Soponaru C., Ilea C., Socolov D.G. (2017). Factors influencing burnout syndrome in obstetrics and gynecology physicians. BioMed Res. Int..

[11] Na’ama O., Naimi A.I., Tulandi T. (2016). Prevalence and predictors of burnout among obstetrics and gynecology residents in Canada. Gynecol. Surg..

[12] Ye J., Wang H., Wu H., Ye L., Li Q., Ma X.Y., Yu X., Zhang H., Luo X. (2019). Burnout among obstetricians and paediatricians: A cross-sectional study from China. BMJ Open.

[13] Kannampallil T.G., Goss C.W., Evanoff B.A., Strickland J.R., McAlister R.P., Duncan J. (2020). Exposure to COVID-19 patients increases physician trainee stress and burnout. PLoS ONE.

[14] Chua M.S.Q., Lee J.C.S., Sulaiman S., Tan H.K. (2020). From the frontline of COVID-19–how prepared are we as obstetricians? A commentary. BJOG Int. J. Obstet. Gynaecol..

[15] Vafaei H., Roozmeh S., Hessami K., Kasraeian M., Asadi N., Faraji A., Bazrafshan K., Saadati N., Aski S.K., Zarean E. (2020). Obstetrics Healthcare Providers’ Mental Health and Quality of Life During COVID-19 Pandemic: Multicenter Study from Eight Cities in Iran. Psychol. Res. Behav. Manag..

[16] Phelan A.L., Katz R., Gostin L.O. (2020). The Novel Coronavirus Originating in Wuhan, China: Challenges for Global Health Governance. JAMA.

[17] WHO (2020). WHO Health Emergency Dashboard WHO (COVID-19) Homepage. https://covid19.who.int/.

[18] National Institute of Statistic (2020). Statistics NIo. Inhabitant population of Romania at 01.01.2020.

[19] Statistics NIo (2020). Coronavirus COVID-19 in Romania. https://covid19geo-spatialorg/.

[20] Pappa S., Ntella V., Giannakas T., Giannakoulis V.G., Papoutsi E., Katsaounou P. (2020). Prevalence of depression, anxiety, and insomnia among healthcare workers during the COVID-19 pandemic: A systematic review and meta-analysis. Brain Behav. Immun..

[21] Zhang C., Yang L., Liu S., Ma S., Wang Y., Cai Z., Du H., Li R., Kang L., Su M. (2020). Survey of insomnia and related social psychological factors among medical staff involved in the 2019 novel coronavirus disease outbreak. Front. Psychiatry.

[22] Marjanovic Z., Greenglass E.R., Coffey S. (2007). The relevance of psychosocial variables and working conditions in predicting nurses’ coping strategies during the SARS crisis: An online questionnaire survey. Int. J. Nurs. Stud..

[23] Mappa I., Distefano F.A., Rizzo G. (2020). Effects of coronavirus 19 pandemic on maternal anxiety during pregnancy: A prospectic observational study. J. Perinat. Med..

[24] Derya Y.A., Altiparmak S., Emine A.K., GÖkbulut N., Yilmaz A.N. (2020). Pregnancy and Birth Planning During COVID-19: The Effects of Tele-Education Offered to Pregnant Women on Prenatal Distress and Pregnancy-Related Anxiety. Midwifery.

[25] Rubin R. (2020). COVID-19’s crushing effects on medical practices, some of which might not survive. JAMA.

[26] Gómez-Salgado J., Domínguez-Salas S., Romero-Martín M., Ortega-Moreno M., García-Iglesias J.J., Ruiz-Frutos C. (2020). Sense of Coherence and Psychological Distress Among Healthcare Workers During the COVID-19 Pandemic in Spain. Sustainability.

[27] Marques I., Serrasqueiro Z., Nogueira F. (2021). Managers’ Competences in Private Hospitals for Investment Decisions during the COVID-19 Pandemic. Sustainability.

